# Ecotypic and genotypic effects on regrowth and heading date in switchgrass (*Panicum virgatum*)

**DOI:** 10.1002/pld3.111

**Published:** 2019-01-10

**Authors:** Qingzhen Jiang, Stephen L. Webb, Hem S. Bhandari, Joe H. Bouton, Malay C. Saha

**Affiliations:** ^1^ Noble Research Institute, LLC Ardmore Oklahoma; ^2^ Department of Plant Sciences University of Tennessee Knoxville Tennessee; ^3^ Crop and Soil Sciences University of Georgia Athens Georgia

**Keywords:** biomass, heading date, regrowth, switchgrass, variance components analysis, vegetative growth period

## Abstract

Switchgrass (*Panicum virgatum* L.) is a native perennial grass species with great potential for bioenergy and forage. However, knowledge about its genetics and biology related to breeding is still in its infancy. Studying the diversity of switchgrass germplasm will shed light on variability, response to environmental conditions, adaptability, breeding, etc. Thirty‐six switchgrass accessions/cultivars were used to study the ecotypic and genotypic effects on regrowth, heading date, and vegetative growth period. The R‐360 honeycomb design was used for planting these accessions in 2007. Data on regrowth and heading dates were recorded in 2008, 2010, and 2011. Vegetative growth period was calculated by subtracting the regrowth date from the heading date. It was found that the lowland started regrowing earlier (77 ± 0.4 days of the year, DOY) than the upland ecotype (82 ± 0.3 DOY). The upland had earlier heading date (160 ± 0.4 DOY) than the lowland ecotype (173 ± 0.5 DOY). Vegetative growth period was about 18 days longer in the lowland (89 ± 0.6 days) than the upland ecotype (71 ± 0.4 days). For switchgrass (i.e., all accessions), biomass yield was related positively to growth period and heading date; however, biomass was only weakly related to regrowth. Therefore, when targeting biomass in the breeding program, growth period may be a quick and reliable reference in both ecotypes to quickly estimate biomass potential while regrowth and heading date may be better used as a parameter for accessions within an ecotype.

## 1 INTRODUCTION

Switchgrass (*Panicum virgatum* L.) is a perennial grass species native to North America. It can be used for hay production, grazing by livestock, or land restoration (Cortese, Honig, Miller, & Bonos, 2010; Nageswara‐Rao, Soneji, Kwit, & Stewart, 2013; Vogel, 2004). Before 1990, breeding effort mostly focused on upland germplasm as forage to improve livestock production (Casler et al., 2011). The U.S. Department of Energy (DOE) selected this species as the herbaceous model for bioenergy feedstock, resulting in greater research into its biology (ecotype, ploidy, genome sequencing, etc.), cultivation, physiology, and breeding for use as a biofuel (Cortese et al., 2010).

Vegetative regrowth is a very important agronomic trait including the conservation of vegetative meristem and storage and remobilization of nutrients in roots (Vriet, Smith, & Wang, 2014). The ability for regrowth in the spring after a severe winter is generally used as an indicator for winter hardiness of turf and forage grasses (Stefaniak, Rodgers, VanDyke, Williams, & Phillips, 2009). In general, perennial grass species develop a winter‐hardy crown containing buds and meristems for tillers and start regrowing when temperature is optimal in spring. In spring, new tillers evolve from the crown and develop as harvestable crop. Unlike annual species, the persistence of perennial species is critical for its overall performance and quality as well as yield (Glover et al., 2010; Wilkins & Humphreys, 2003). Persistence refers to a positive net balance between new production and loss of a species/cultivar over the years of growth by vegetative (tillering or branching) or reproductive (natural reseeding) procedures or both (Nie, Chapman, Tharmaraj, & Clements, 2004). Overall, early regrowth is a favorable trait under warmer winter‐spring climate conditions with possible longer vegetative growth period.

However, perennials with early regrowth characteristics may encounter adverse weather (e.g., frosts in early spring) and be killed or damaged for continuous vigorous growth in the spring. Cultivars belonging to lowland ecotype of switchgrass are more susceptible to this damage compared to upland ecotypes in Ardmore, Oklahoma (personal communication, Saha). In perennial ryegrass, cultivars with early regrowth in the spring tended to be winter hardy (Yu et al., 2015). Late regrowth, on the other hand may be a defensive mechanism to avoid late frost in the spring. However, it leaves a shorter period for vegetative growth before summer drought or transition to reproductive stage. Therefore, a species must adapt to survive and reproduce within its environment (Chapman, Lee, & Waghorn, 2014). During the vegetative growth period, leaves are developed and this is the best stage for livestock feed. Therefore, longer vegetative growth period (i.e., longer time for light harvest and photosynthesis) is beneficial for feed quality and favors for selection as forage yield as well as biomass for biofuel because of its low lignin content.

Reproductive development (characterized by heading or flowering) of grasses generally reduces the distribution of dry matter into leaves while increasing it to stems (Chapman et al., 2014) and starting lignification. In addition, the digestibility of the plant declines due to fibrousness and palatability of the stems with shed leaves. Hence, late heading date with early regrowth, or more specifically long vegetative growth period is promising in selection for both forage quality as well biomass for biofuel in switchgrass. However, few studies have focused on regrowth, heading date, and vegetative growth period in switchgrass, with a couple of exceptions (Bhandari, Saha, Fasoula, & Bouton, 2011; Bhandari, Saha, Mascia, Fasoula, & Bouton, 2010).

Genetic diversity is the basis for breeding programs. Breeding programs may be accelerated by characterizing genetic variation from available germplasm and by maximizing useful genetic variation in breeding populations (Glover et al., 2010). Collections of switchgrass germplasms with large genetic diversity have become a common effort for many programs including both public and private sectors (Casler et al., 2011). Using collections of germplasms from diverse environments is a way to develop the potential for broad diversity of genetic resources. In the current study, 36 switchgrass accessions, representing 18 U.S. states and three countries, were evaluated for their growth performance including spring regrowth, heading date, and vegetative growth period. The objectives were to: (a) investigate the differential performance of these accessions in agronomically important traits such as spring regrowth and heading date, (b) evaluate the relationships between biomass yield and these traits so that earlier selection may be possible, and (c) select accessions with potential for improving switchgrass as forage as well as bioenergy crop through these growth‐related traits.

## 2 MATERIALS AND METHODS

### 2.1 Plant materials and resources

Among the 36 switchgrass accessions (Jiang et al., 2014; Table S1) studied, seeds of 31 plant introduction (PI) lines were obtained from the USDA‐Germplasm Resources Information Network collected from 18 different U.S. states and three international countries including Argentina, Belgium, and Turkey. The other five were breeding populations and released cultivars. Ten genotypes per accession were evaluated, resulting in 360 total genotypes (i.e., samples).

### 2.2 Experiment site and design

The experimental site was located at the Noble Research Institute, Research Park (34°11′N, 97°05′W, 266 m a.s.l.). The soil type was Normangee clay loam (fine, smectitic, thermic Udertic Haplustalfs) with mean pH = 5.7. Four clonal replicates from each genotype were transplanted in the field on 1 August, 2007 according to an R‐360 honeycomb design (Fasoulas & Fasoula, 1995) (see Jiang et al., 2014 for details).

### 2.3 Trait measurement and procedure

Data on regrowth and heading dates were collected from each of the 10 genotypes of all accessions and replications. Spring regrowth was calculated as ordinal days (i.e., days of year, DOY) after 1 January (1 DOY) to first shoot growth. The entire experimental field was scouted every other day for recording any visible new shoot regrowth in each genotype clump. Similar scouting was conducted during the heading time; Days from 1 January to the date of first head emerging completely from the flag leaf sheath was recorded as heading date. Days from spring regrowth to heading were considered as active vegetative growth period. Biomass was harvested separately for each individual plant after killing frost in each year.

### 2.4 Statistical analysis

Data were analyzed using generalized linear mixed models (GLMM) and restricted maximum likelihood estimators in the GLIMMIX procedure of SAS^®^ 9.3 (SAS Institute, Inc., Cary, NC) (Littell, Milliken, Stroup, Wolfinger, & Schabenberger, 2006). A hierarchical approach was used for data analysis. Variance components for the full array of 36 switchgrass accessions were first analyzed to make inferences on switchgrass at the crop level. Next, we also modeled ecotype‐specific variance components where accessions were analyzed within each ecotype (lowland and upland). The three traits of interest were regrowth date, heading date, and growth period. Details on the data analysis can be obtained from Jiang et al., 2014.

Generalized linear mixed models were used to assess mean ecotype differences in switchgrass for regrowth, heading, and growth period where ecotype was the main effect with two levels (lowland and upland). Four random effects (accession, accession × genotype, year and year × accession) were modeled to also partition the sources of variation in each of the three traits. Throughout, variance components covariance structure was used and the Kenward‐Rogers denominator degrees of freedom adjustment (Kenward & Roger, 1997) was specified to account for unbalanced data and multiple random effects (Littell et al., 2006). Next, each ecotype was analyzed separately to examine mean accession differences for the three traits and for further partitioning variation at this level, where accession was the main effect and genotype, year, and genotype × year were modeled as random effects.

From the variance component analysis, broad‐sense heritability for each trait also was calculated for each level of analysis (Falconer & Mackay, 1996). Broad‐sense heritability estimates were carried out following the method described in Jiang et al. (2014). Estimated heritability values close to 0 indicated strong environmental (i.e., year in the current study) influence, whereas values near 1 showed a strong genetic control (Lynch & Walsh, 1997).

Last, relationships also were examined between regrowth date, heading date, and growth period with biomass. If a relationship exists between any of the three traits and biomass, then regrowth date, heading date, or growth period could be used as a surrogate to estimating biomass yield. The REG procedure within SAS^®^ 9.3 was used to determine whether regrowth date, heading date, and growth period (independent variables) were related to biomass yield (dependent variable). A full description of biomass yield data can be obtained from Jiang et al. (2014). Before analysis, biomass was log‐transformed to correct for lack of homogeneity of variance and normality of distribution. Regression analyses were run for all 36 accessions to assess relationships between traits for switchgrass in general. Regression analysis also was run for each ecotype separately. All means were reported ± SE throughout.

## 3 RESULTS

### 3.1 Earlier regrowth was observed in the lowland than upland accessions

**Figure 1:**
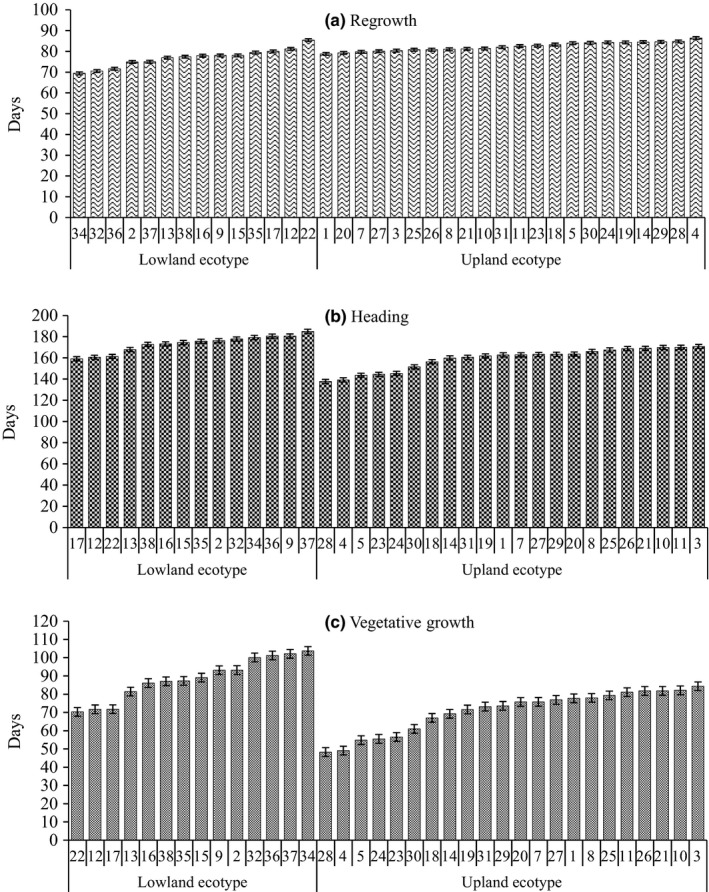
Distribution of regrowth (a), heading dates (b) and vegetative growth period (c) in switchgrass populations evaluated at Ardmore, OK during 2007–2011. Error bars show standard error of the populations

The overall regrowth date for the studied 36 switchgrass accessions was 80 ± 0.2 DOY (mean±SE). In general, the lowland accessions started regrowth earlier (77 ± 0.4 DOY) than upland accessions (82 ± 0.3 DOY) (*F*_1,34.0_ = 25.12, *P *<* *0.001). Large differences in regrowth date were observed among the accessions in each ecotype. In the lowland accessions, regrowth started from 69 ± 0.9 (EG 1101) to 85 ± 1.0 (T 2101) DOY (*F*_13,1321_ = 58.72, *P *<* *0.001) (Table 1). EG1101 together with Alamo and GA991 are in a group that had the earliest regrowth date. These cultivars/accessions were bred or adapted to the south‐central US with warm conditions. The other lowland accessions were comparable to each other in their regrowth date except T 2101 which was collected from New Jersey. T 2101 had the regrowth date (85 ± 1.0 DOY) later than most of the upland accessions. In the upland accessions, regrowth started from 79 ± 0.9 (T 2100) to 86 ± 0.9 (156) DOY (*F*_21,2268_ = 24.04, *P *<* *0.001) (Table 1, Figure 1). The differences within the upland accessions are narrower than the lowland populations except accession 156 which was collected from Belgium. Accession 156 had the latest regrowth date.

**Table 1:** Means (±SE) for growth‐related characteristics (regrowth date, heading date and growing period) of 36 switchgrass accessions averaged across 3 years (2008, 2010, and 2011) in south‐central Oklahoma, USA. Data were analyzed using generalized linear mixed models in SAS^®^ 9.3

Ecotype	Code	Accession	Regrowth (DOY)	Heading (DOY)	Vegetative growth period (days)
Lowland	2	BN‐14668‐65	75 ± 1.1	176 ± 1.2	93 ± 1.3
	9	Kanlow	78 ± 1.3	181 ± 1.0	93 ± 1.0
	12	BN‐13645‐64	81 ± 1.2	161 ± 0.9	72 ± 1.2
	13	T 2099	77 ± 1.8	168 ± 1.8	81 ± 1.7
	15	BN‐14669‐92	78 ± 1.2	174 ± 1.2	89 ± 1.2
	16	BN‐8358‐62	78 ± 1.3	173 ± 0.9	86 ± 0.9
	17	BN‐11357‐63	80 ± 1.2	159 ± 0.6	72 ± 0.8
	22	T 2101	85 ± 1.0	161 ± 1.3	70 ± 1.4
	32	Alamo	71 ± 1.5	178 ± 2.5	100 ± 2.6
	34	EG1101	69 ± 0.9	179 ± 1.3	104 ± 1.5
	35	Tennessee	79 ± 1.5	176 ± 1.0	87 ± 1.2
	36	GA991	72 ± 1.2	181 ± 1.1	101 ± 1.3
	37	EG1102	75 ± 1.1	185 ± 1.1	102 ± 1.0
	38	AP13 × VS16	77 ± 1.4	173 ± 1.0	87 ± 1.6
Upland	1	T 2100	79 ± 0.9	163 ± 0.7	78 ± 0.9
	3	196	80 ± 0.9	171 ± 0.9	84 ± 0.9
	4	156	86 ± 0.9	139 ± 1.1	49 ± 1.1
	5	T 4613	84 ± 0.8	144 ± 1.1	55 ± 1.2
	7	Cave‐in‐Rock	80 ± 1.0	163 ± 0.8	76 ± 0.8
	8	Blackwell ‐KS	81 ± 1.0	166 ± 1.0	78 ± 1.0
	10	BN‐10860‐61	81 ± 0.9	170 ± 0.9	82 ± 1.0
	11	KY 1625	83 ± 0.9	170 ± 0.7	81 ± 0.7
	14	Cen. Iowa Germp.	84 ± 0.9	160 ± 1.0	69 ± 1.0
	18	70SG 001	83 ± 0.9	156 ± 1.5	67 ± 1.3
	19	70SG 024	84 ± 0.9	162 ± 1.0	72 ± 1.0
	20	Shawnee	79 ± 1.2	164 ± 0.6	75 ± 0.6
	21	Trailblazer	81 ± 0.9	169 ± 1.0	82 ± 1.0
	23	Grenville	83 ± 0.8	145 ± 1.2	57 ± 1.2
	24	Falcon	84 ± 0.9	145 ± 1.1	56 ± 1.2
	25	BN‐309‐69	81 ± 1.0	168 ± 0.8	79 ± 0.9
	26	Blackwell ‐ OK	81 ± 0.9	169 ± 0.6	82 ± 0.6
	27	Caddo	80 ± 0.9	163 ± 1.0	77 ± 1.0
	28	Dacotah	85 ± 1.0	138 ± 1.1	48 ± 1.3
	29	Summer	85 ± 1.0	163 ± 1.0	74 ± 1.2
	30	Sunburst	84 ± 0.9	152 ± 1.3	61 ± 1.2
	31	Ankara	82 ± 0.8	161 ± 0.7	73 ± 0.7

Among the variance components in the 36 switchgrass for regrowth dates, year contributed the most, explaining 79% of the variation (Table 2). Accession in addition to its interaction with year or genotype contributed lower percentages (4.2%, 3.9% and 2.4%, respectively) of the variance. Heritability of regrowth is very low in switchgrass (*H* ^2^ = 0.07) (Table 2). When the 36 accessions were separated according to ecotype, year accounted for the largest variation in regrowth of both ecotypes (86% and 87%, respectively) (Table 3). Genotype (0.3% and 0% for lowland and upland) and its interaction with year (0.3% and 0% for lowland and upland) had little effect on the variance in regrowth (Table 3).

**Table 2:** Variance component analysis and broad‐sense heritability of the growth‐related traits of switchgrass (across 36 accessions from lowland and upland ecotypes) over 3 years (2008, 2010 and 2011) in south‐central Oklahoma, USA. Values represent covariance parameter estimates followed by percentage contribution (in parentheses) of each variance component to each trait

Component	Regrowth	Heading	Growth period
Accession	6.8 (4.2)	83 (46)	113 (52)
Genotype (Accession)	3.9 (2.4)	29 (16)	30 (13)
Year	127 (79)	13 (7.2)	0 (0)
Accession × Year	6.2 (3.9)	13 (7.1)	23 (10)
Residual	17 (10)	43 (24)	55 (25)
Total	160	180	220
Heritability (*H*²)	0.07	0.62	0.65

**Table 3:** Variance component analysis of the growth‐related traits for lowland and upland ecotypes of switchgrass over 3 years (2008, 2010 and 2011) in south‐central Oklahoma, USA. Values represent covariance parameter estimates followed by percentage contribution (in parentheses) of each variance component to the corresponding trait

Component	Lowland			Upland		
	Regrowth	Heading	Growth period	Regrowth	Heading	Growth period
Genotype	0.7 (0.3)	0 (0)	0 (0)	0 (0)	3.1 (4.1)	3.0 (3.8)
Year	189 (86)	35 (33)	15 (12)	123 (87)	3.8 (5.0)	1.5 (1.9)
Genotype × Year	0.6 (0.3)	3.4 (3.1)	2.7 (2.2)	0.1 (0)	2.4 (3.1)	3.1 (3.9)
Residual	29 (13)	70 (64)	104 (86)	18 (13)	67 (88)	71 (90)

### 3.2 The upland had earlier heading than the lowland accessions

The heading date of 36 accessions varied from 185 ± 1.1 (EG1102) to 138 ± 1.1 (Dacotah) DOY with the mean of 165 ± 0.3 DOY. The upland accession (160 ± 0.4 DOY) was about 13 days earlier in heading than the lowland accession (173 ± 0.5 DOY) (*F*_1,34.1_ = 18.52, *P *<* *0.001). The heading date in the lowland accessions ranged from 159 ± 0.6 (BN‐11357‐63) to 185 ± 1.1 (EG1102) DOY and differed across accessions (*F*_13,932.2_ = 66.69, *P *<* *0.001). Heading date in the upland ranged from 138 ± 1.1 (Dacotah) to 171 ± 0.9 (Accession 196) DOY, which was statistically different across accessions (*F*_21,1654_ = 115.19, *P *<* *0.001) (Table 1, Figure 1).

Variance components for heading were distributed differently than for regrowth. For heading date, accession (46%) played the most important role in determining its value, followed by the interaction between accession and genotype (16%) (Table 2). Year and its interaction with accession contributed 7.2% and 7.1% to the total variance of heading. When the 36 accessions were separated according to ecotype, the significance of the components was quite different. For lowland, year accounted for 33% of the variance; however, for upland, year only accounted for 5.0% of the variance (Table 3). For heading date, the heritability was relatively high (*H* ^2^ = 0.62) owing to the major contribution from accession (Table 2).

### 3.3 The lowland had longer vegetative growth period than the upland accessions

The vegetative growth period was calculated from the heading date and regrowth date. As a result, the growth period was longer within the lowland (89 ± 0.6 days) than upland (71 ± 0.4 days) accessions (*F*_1,34.1_ = 20.94, *P *<* *0.001); the overall mean vegetative growth period was 78 ± 0.3 days for all accessions combined. The vegetative growth period had a range of 70 ± 1.4 to 104 ± 1.5 days within the lowland (*F*_13,920.2_ = 86.06, *P *<* *0.001) and of 48 ± 1.3 to 84 ± 0.9 days within the upland (*F*_21,1635_ = 125.52, *P *<* *0.001) (Table 1, Figure 1). The three lowland accessions BN‐11357‐63, T 2101 and BN‐13645‐64 had the latest regrowth date but the earliest heading and thus shortest growth period even shorter than some of the upland accessions. Among the upland accessions, 156 and Dacotah had latest regrowth date but earliest heading and thus shortest growth period (Figure 1).

The variance component analysis of switchgrass in general indicated that accession (52%) had the strongest effect on the growth period followed by accession×genotype interaction (13%) (Table 2). The interaction between accession and year contributed 10% of the variance in the growth period while year had no effect (Table 2). Different contribution patterns were noticed when ecotype was considered. Genotype had no effect on vegetative growth period in the lowland and only accounted 3.8% of the variance in the upland ecotype. For the growth period, the heritability in switchgrass was high (*H* ^2^ = 0.65) because of the large contribution from accession (Table 2).

### 3.4 Relationships among growth traits and biomass

Using 36 accessions including lowland and upland ecotypes, this relationship was investigated combining all the accessions as well as within each ecotype. For switchgrass as a whole, regrowth date had a weak negative relationship (−0.02 ± 0.01 SE) with biomass (*R*² = 0.04, *P *=* *0.035) that may not be valuable in reality. However, heading date (0.05 ± 0.008 SE; *R*² = 0.36, *P *≤* *0.001) and growth period (0.05 ± 0.006 SE; *R* ^2^ = 0.55, *P *≤* *0.001) were positively related with biomass.

When ecotype was considered separately, a weak positive relationship was only found between regrowth and biomass yield in the lowland accessions (*R* ^2^ = 0.10, *P *=* *0.04) while the relationship was not significant in the upland accessions (*R* ^2^ = 0.02, *P *=* *0.32).

**Figure 2:**
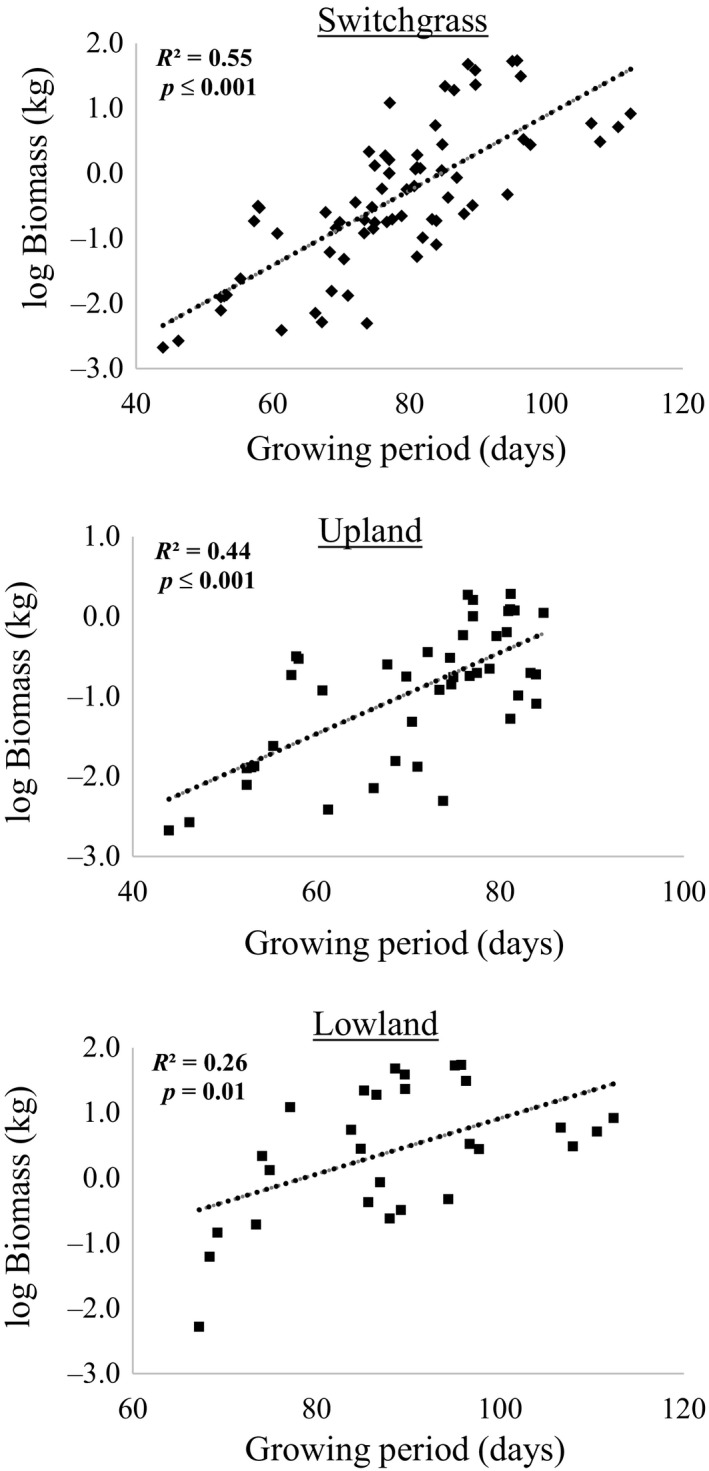
Linear regression of the biomass yield over growth period for all 36 switchgrass accessions, and over upland and lowland ecotypes, respectively

Conversely, for heading with biomass, the relationship was stronger within the upland accessions (*R*² = 0.24, *P *<* *0.001) but it was not significant within the lowland accessions (*R* ^2^ = 0.07, *P *=* *0.16). When biomass was regressed over growth period within each ecotype, growth period had more effect on biomass yield in the upland (*R* ^2^ = 0.44, *P *<* *0.001) than the lowland accessions (*R* ^2^ = 0.26, *P *=* *0.01) (Figure 2).

Additionally, when considering all switchgrass accessions, a negative correlation was observed between heading/growth period and regrowth date (*r *=* *−0.24, *P *<* *0.001; *r *=* *−0.54, *P *<* *0.001, respectively) indicating that selecting for one trait (heading or growth period) may have to compensate for another trait (regrowth date).

## 4 DISCUSSION

Traditionally switchgrass has been used as forage and in conservation plantings. Recently, it has been selected as a dedicated feedstock for the biofuel production by the U.S. DOE. High biomass yield is the target trait for any switchgrass breeding program. Yield is a complex trait contributed by several co‐related traits. Switchgrass is a perennial grass species. It senesces at the end of a growing season, remains dormant during the winter, and starts regrowing in the spring. At heading, vegetative growth slows down and the plants enter reproductive phase. Thus the length of vegetative growth period, i.e. the period between regrowth and heading, is an important characteristic, which largely determines the biomass production potential of switchgrass. Three important traits of switchgrass were studied including regrowth, heading, and vegetative growth period and their relationship with biomass yield. Relationships between these traits could be exploited in an effective breeding program to redesign switchgrass for optimal performance/yield.

### 4.1 Broad range was observed in regrowth date

Spring regrowth was recorded from 1 January to the first day of greening of the plants. After winter, as soon as the soil temperature becomes favorable, switchgrass starts regrowing. Wide variability was observed in the 36 accessions for their ability to start regrowing after winter (69‐86 DOY). In general, the lowland ecotypes started regrowing earlier than the upland accessions. Selection for high biomass yield could have selected plants that break out dormancy early in the spring. Bhandari et al. (2010) reported that the EG 1101 and Alamo started regrowth on 63 and 64 DOY, respectively. These numbers are comparable with what was found in the current study. The little discrepancy might be related with the different weather conditions during the years that the experiments were conducted. A trend has been observed that the accessions originated from the northern latitudes started regrowing later in the spring than the accessions originated from the southern latitudes. In addition, accessions from the eastern USA may have growth restricted by the drier and windier environments in the Great Plains (Cornelius & Johnston, 1941). In the lowland half‐sib families selected for southern Oklahoma and Texas, the mean spring regrowth date was 66 DOY (range: 59–74 DOY) (Bhandari et al., 2010) while the 46 full‐sib families originated from the same population had the spring regrowth date of 60.7 DOY (range: 53.4–72.7 DOY) (Bhandari et al., 2011). Earlier regrowth in full‐sib families compared to half‐sib families could have resulted from unintended biases in making crosses, because genotypes were crossed as they were flowering; and some of the late flowering genotypes could have been left out (HS Bhandari, personal communication).

Among the upland accessions, 156 had the latest regrowth date, even later than the lowland accessions (i.e., T2101, BN‐13645‐64, BN‐11357‐63). This may be explained by its origin with the colder temperature as well as longer photoperiod requirement during growing season for this accession. Overall, except those accessions mentioned earlier, some lowland accessions had similar regrowth date as the upland accessions indicating that this trait is not closely linked genetically with ecotype itself. In cold weather conditions, early regrowth might bring the accessions into a harsh condition and be killed by the extreme temperature during early spring. Planting late regrowing lowland accessions with later regrowth date like BN‐11357‐63, T 2101, and BN‐13645‐64 may have potential to be adaptive to such climate for higher biomass yield than the upland accessions.

Variance components analysis and heritability could provide estimates of genetic variability for quantitative traits (Beyene, Botha, & Myburg, 2005). Since year was the major player in affecting the regrowth date, it is quite straightforward that heritability of regrowth is very low in switchgrass. The smaller variance due to accessions in both ecotypes also demonstrated low heritability of regrowth. However, when only additive genetic effects are considered, narrow sense heritability of spring regrowth was noticed consistently high in the lowland half‐sib families (Bhandari et al., 2010). Among other factors i.e., environment, the reason for this difference in heritability estimates may be due to less genetic background diversity compared to those used in the current study. Variance components analysis in each ecotype showed that regrowth may not be a good indicator to select accessions within an ecotype.

### 4.2 Variation in heading date indicates inhomogeneous genetic background

At heading, reproductive development of perennial grasses became most active. Distribution of dry matter generally reduces in leaves but increases into stems (Chapman et al., 2014). Lignification increases with maturation and thus the quality of feedstock or forages declines after heading due to reduced degradability of fiber in both switchgrass and big bluestem (*Andropogon gerardii*) (Jung & Vogel, 1992). Heading date in the 36 accessions varied from 138 to 185 DOY. It is possible that some of the switchgrass accessions can maintain high feedstock and/or forage quality for a longer time than the others. This variation in heading dates also influences the productivity of the accessions.

The upland accessions were earlier in heading (160 ± 0.4 DOY) than the lowland accessions (173 ± 0.5 DOY), which was consistent with previous findings that heading date was earlier in germplasm from more northern areas/upland than those from southern parts/lowland (Cornelius & Johnston, 1941; Cortese & Bonos, 2013; Eberhart & Newell, 1959; Hopkins, Vogel, Moore, Johnson, & Carlson, 1995a,b). Upland ecotype is adaptive to the northern part with longer photoperiod during growing season. Relatively short day affected their response for reproductive development (heading) (Cornelius & Johnston, 1941). It is interesting to see that all of the lowland accessions used in the current study had earlier heading date than the lowland half‐sib (214‐236 DOY) and the full‐sib (218.2‐235.8 DOY) families bred specifically for the Oklahoma and Texas regions (Bhandari et al., 2010, 2011). It is possible that the lowland accessions used by Bhandari et al. had more homogeneous genetic background and specifically selected only late heading types in their study. The large range of values in the heading date of these 36 accessions indicates the potential for selection and biomass production in the field (Fike, Butler, & Mitchell, 2014).

Medium to high heritability of heading date was observed in many monocot species (Akinwale et al., 2011; Ehdaie & Waines, 1989; Fang, Aamlid, Jørgensen, & Rognlim, 2004; Vogel, Gorz, & Haskins, 1981). Moderate heritability of the accessions used in the current study indicates that heading date could be applied as phenotypic selection in switchgrass breeding program. However, because of the high contribution from factors other than genotype, the heritability of this trait was low in both ecotypes showing the weak reliability for selection within ecotype. For the heading date possible role of non‐additive gene or epistatic gene effects was reported in switchgrass (Bhandari et al., 2011). Several full‐sib families demonstrated positive heterosis for days to heading (up to 31%), which means that families were late in heading compared to the average of their parents.

### 4.3 Vegetative growth period is influenced by both genetics and environment

Vegetative growth period is an important characteristic of grass species. Longer vegetative growth period is related to additional harvest of photosynthetic products, which can be utilized by the plants for better biomass production. The vegetative growth period varied from 48 to 104 days in the 36 switchgrass accessions. This justifies the wide variability among the genotypes for biomass production. On an average, the lowland accessions had 18 days additional vegetative growth period compared to their upland counterparts. The long vegetative period could partially explain the superior biomass yield performance in lowland accessions in general. When grown in the Northeastern/Mid‐Atlantic region, Alamo, Kanlow, and Cave‐in‐Rock could have their heading dates as late as 208.1, 205.1, and 183.1 DOY, respectively (Cortese & Bonos, 2013), indicating that cooler climatic conditions have lengthened their vegetative growth period. This may be the reason for superior biomass production of these cultivars in the region. However, susceptibility to cold weather is a major limitation of growing lowland ecotypes in the North.

Accession is identified as the most powerful component and year as the least in variance components analysis. Vegetative growth period has the highest heritability among the studied traits. Variance component analysis at the ecotypic level failed to partition the variance into main components (genotype, genotype×year) and thus residual absorbed more than 86% of the total variances. High heritability of vegetative growth period indicates the possibility to use the trait for selection. However, due to the major effect from factors other than genotype, the heritability of vegetative growth period was very low in both ecotypes indicating that this trait is not reliable for selection within ecotype.

### 4.4 Relationships among growth traits and biomass indicate possibility as reference for biomass in switchgrass

Even though biomass is a common trait to be targeted in breeding program, regrowth, or heading date may be useful to help make the selection earlier to accelerate the procedure for breeding efforts. The foundation for this hypothesis is a strong relationship between these traits and biomass yield in switchgrass. If used for grazing, earlier regrowth could be a favorable trait so that switchgrass may be used earlier while other forage species are in short provision. Positive relationship was found between biomass yield and heading date among 10 switchgrass populations in New Jersey (Cortese & Bonos, 2013). Early maturity was often related with low forage yield when 23 accessions were studied (Hopkins et al., 1995b). However, no significant associations were found between heading date and yield when 20 elite switchgrass populations were used (Hopkins et al., 1995a) indicating that selection pressure has successfully dissected these traits in the breeding procedure.

Substantial positive relationship was observed between heading date and biomass within the upland accessions (*R* ^2^ = 0.24, *P *=* *0.001) while it was not significant within the lowland accessions (*R* ^2^ = 0.07, *P *=* *0.16). Even using lowland half‐sib family with relatively more homogeneous genetic background, the correlations between dry matter yield and spring regrowth as well as dry matter yield and days to heading were not significant (Bhandari et al., 2010). Therefore, in lowland populations, heading date may be selected independently without affecting biomass yield.

A strong positive relationship was found between biomass and vegetative growth period in the 36 accessions (*R* ^2^ = 0.55, *P *<* *0.001). This was easy to interpret because with longer growth period more solar energy is harvested for photosynthesis and more carbon is fixed in the plants for biomass production. When breeding for biomass in some populations, growth period can be a valuable trait to select. Accessions with later heading are thus more desirable for biomass production since the growth period is generally longer (Cortese & Bonos, 2013; Sripathi, Kakani, & Wu, 2013). However, in colder regions, winter survival may need more attention for perennial crops than growth period because late maturing accessions are generally more susceptible to freezing temperature (Cortese & Bonos, 2013). On the other hand, in areas in which freezing temperature is not a concern (e.g., Oklahoma), accessions with early regrowth and late heading would have longer growth period that will be favorable for higher biomass yield.

At ecotypic level, growth period has more effect on biomass yield in the upland than lowland accessions. In all situations, shorter growth period brought up lower biomass yield in general. This may be the major reason that lowland switchgrass usually has higher biomass yield than upland. Considering breeding target for biomass, accessions from lowland accessions should be the first choice; on the other hand, the upland accessions may be used to screen some cold‐related mechanisms so as to be integrated into the lowland accessions for improvement in cold tolerance. In all, when selecting cultivar with high biomass promise, local growing conditions should always be considered to maximize its potential (Fike et al., 2014). Accessions with similar morphology or similar genetic background may be helpful for selecting parents to make crosses (Beyene et al., 2005).

## 5 CONCLUSIONS

Switchgrass has brought tremendous attention since 1990 because of its potential use for forage and bioenergy as well as its hardy growth in marginal land. By investigating 36 accessions collected from a wide area, we hoped to uncover relationships between total biomass and several other growth parameters that could provide insight into improving switchgrass’ value both as forage and for bioenergy. Broad diversity existed in these accessions indicating that selection of lines with contrasting genetic background is possible to improve their persistence to adapt to different climatic conditions. In general, in the southern regions, the lowland accessions may be more focused for feedstock production while the upland accessions have limited application for biofuel production because of their poor performance (low regrowth, early heading date, and short vegetative growth period). Accessions with earlier growth date, e.g., EG1101, could be considered for forage breeding to compensate the shortage of feed in early spring. In addition, longer vegetative growth period could provide longer grazing time as well as high potential for biomass. Accessions like EG1101, EG1102, GA991, and Alamo are the best candidates that have potential to grow in this region as forage feed as well as bioenergy crop. Besides genetic effects, regrowth and heading date are also related with temperature and day length. Further research will focus on identifying QTLs associated with these traits as well as environmental effects that may provide further support for cultivar improvement through molecular breeding approaches.

## AUTHORS CONTRIBUTIONS

QJ organized and analyzed the data, interpreted the results and developed the draft of the manuscript. SW performed data analysis, interpreted the data, and contributed significantly in drafting the manuscript. HB helped in field experimentation, data organization, and drafting and editing the manuscript. JB helped in designing the experiment, supervise project activities and contributed in editing the manuscript. MS conceived, supervised the whole project, and contributed to the drafting and editing of the manuscript.
