# Supplementary material for: Ecotypic and genotypic effects on regrowth and heading date in switchgrass (Panicum virgatum)
Source: Plant Direct. 2019 Jan 10;3(1):e00111. doi: 10.1002/pld3.111 (PMC6508767; doi:10.1002/pld3.111)
Supplement: Supplementary file 1 [file PLD3-3-e00111-s001.pdf]

Supplementary materials:

Supplementary Table 1. Switchgrass accessions (Acc.): their source of collection, PI/cultivar status, and ecotype classification based on SSR and chloroplast DNA marker analysis

Acc. #	Germplasm resources	Source	PI/ Cultivar	Eco/Gen SSR[§]	Eco/ Chl[¶]	Citations
1	T 2100 - Arkansas	GRIN*	PI 476292	U	U	
2	BN-14668-65 - Arkansas	GRIN	PI 414065	L	L	
3	196 - Argentina	GRIN	PI 337553	U	U	
4	156 - Belgium	GRIN	PI 442535	U	U	
5	T 4613 - Colorado	GRIN	PI 476294	U	U	
7	Cave-in-Rock - Illinois	GRIN	PI 469228	U	U	Anderson & Sharp, 1994
8	Blackwell - Kansas	GRIN	PI 657663	U	U	Anderson & Sharp, 1994
9	Kanlow - Kansas	GRIN	PI 421521	L	L	Anderson & Sharp, 1994
10	BN-10860-61 - Kansas	GRIN	PI 315724	U	U	
11	KY 1625 - Kentucky	GRIN	PI 431575	U	U	
12	BN-13645-64 - Maryland	GRIN	PI 315728	L	L	
13	T 2099 - Maryland	GRIN	PI 476291	L	L	
14	Cen. Iowa Germp. - Missouri	GRIN	PI 657660	U	U	
15	BN-14669-92 - Mississippi	GRIN	PI 315725	L	L	
16	BN-8358-62 - North Carolina	GRIN	PI 315723	L	L	
17	BN-11357-63 – North Carolina	GRIN	PI 315727	L	L	
18	70SG 001 - North Dakota	GRIN	PI 642193	U	U	
19	70SG 024 - North Dakota	GRIN	PI 642215	U	U	
20	Shawnee - Nebraska	GRIN	PI 591824	U	U	Vogel et al. 1997
21	Trailblazer - Nebraska	GRIN	PI 549094	U	U	Vogel et al. 1991
22	T 2101 - New Jersey	GRIN	PI 476293	L	L	
23	Grenville - New Mexico	GRIN	PI 657664	U	U	Anderson & Sharp, 1994
24	Falcon - New Mexico	GRIN	PI 642190	U	U	
25	BN-309-69 - New York	GRIN	PI 414069	U	U	
26	Blackwell - Oklahoma	GRIN	PI 421520	U	U	Blade Energy Crops
27	Caddo - Oklahoma	GRIN	PI 476297	U	U	Hein 1958
28	Dacotah - Oregon	GRIN	PI 537588	U	U	Barker et al, 1990
29	Summer - South Dakota	GRIN	PI 642191	U	U	
30	Sunburst - South Dakota	GRIN	PI 598136	U	U	Boe and Ross, 1998
31	Ankara, Turkey	GRIN	PI 204907	U	U	
32	Alamo - Texas	GRIN	PI 422006	L	L	
34	EG1101	Ceres	Cultivar	-	L	Blade Energy Crops
35	Tennessee	Noble	Population	-	L	
36	GA991	Noble	Population	-	L	
37	EG1102	Ceres	Cultivar	-	L	Blade Energy Crops
38	AP13 × VS16	Noble	Population	-	L	

[§] Ecotype identified by SSR markers (Narasimhamoorthy et al., 2008)
[¶] Ecotype identified by chloroplast DNA markers
*GRIN= Germplasm Resources Information Network, USDA-ARS; Ceres = Ceres Inc., CA; Noble = Noble Foundation, Ardmore, OK
