# Supplementary material for: Ecotypic and genotypic effects on regrowth and heading date in switchgrass (Panicum virgatum)
Source: Plant Direct. 2019 Jan 10;3(1):e00111. doi: 10.1002/pld3.111 (PMC6508767; doi:10.1002/pld3.111)
Supplement: Supplementary file 2 [file PLD3-3-e00111-s002.pdf]

Response to reviewers for Plant Direct

Dear Editor,

First of all, we thank all the reviewers and the editor for spending their valuable time reviewing our manuscript. We appreciate their comments and suggestions to improve the manuscript.

We revised the manuscript as per suggestions of the reviewers. Please find the revised version with Tracks and Changes. Thank you,

Qingzhen

-- Reviewer comments:

Reviewer #1:

This manuscript describes field experiments seeking to derive relationships between biomass, regrowth, heading, and vegetative period in switchgrass. On a whole the conclusions are well supported by the data. I would require some clarification regarding the data analysis with respect to partitioning variance components between accessions vs. genotypes (see comment below). Why are Tables 2 and 3 treated differently with regard to these. It appears that the terms are not being used consistently correctly. Julian day is acceptable, however DOY, to me, sounds better.

- *Variance component analysis was performed at two levels: i) switchgrass as a whole (across 36 accessions from lowland and upland ecotypes), Table 2, and ii) independently for each ecotype, Table 3.*
- *We consistently use DOY and accessions throughout the manuscript*

Also please address the following:
pg 8 line 149: insert "the lowland accessions" or "the upland accessions" as required throughout manuscript.
- *Modified as suggested*

pg 6 line 101: insert "ordinal days after 1 January (DOY)"
- *Modified as suggested*

pg 6 line 104 insert "DOY at first head emerging"
- *Modified as suggested*

pg 8 line 148/149 insert "on DOY 80 ... ES). In general..." and use DOY throughout as required.
- *Modified as suggested*

pg 8 line 153 "are in a group of south-central US cultivars/accessions. Move "and selection for high...spring." to discussion.
- Modified as suggested

line 174 change to "The heading DOY"
- Modified as suggested

line 181 change to "Variance components for heading were distributed differently than for regrowth.
- Modified as suggested

line 194 change ecotype to accessions.
- Modified as suggested

line 203 change to "followed by accession x genotype interaction"
- Modified as suggested

line 247 suggest changing sentence to "Relationships between these traits could be exploited in an effective breeding program to redesign switchgrass for optimal performance/yield."
- Modified as suggested

line 365 suggest clarifying what was the relationship.
- Modified as suggested

line 396 suggest changing to "as well as its hardy growth in marginal land." and clarifying the following sentence starting with "By investigating..." Perhaps "By investigating 36 accessions collected from a wide area, we hoped to uncover relationships between total biomass and several other growth parameters that could provide insight into improving switchgrass' value both as forage and for bioenergy."
- Modified as suggested

Table 1: Change Julian days to DOY.
- Modified as suggested

In Table 2 and Table 3 clearly indicate which variance components apply to genotype or Accession and explain the reason for the difference.
- Variance component analysis was performed at two levels: i) switchgrass as a whole (across 36 accessions from lowland and upland ecotypes) and ii) independently for each ecotype. We clarified this in the materials and methods and table captions are modified accordingly.

Please refer to Supplemental table in Materials and Methods section.
- Supplemental table referred

Reviewer #2:

This manuscript examined the ecotypic and genotypic effects on regrowth and heading date in switchgrass (Panicum virgatum). The topic is of interest which may attract a broad range of readers. The experiments were designed and conducted in an appropriate manners. I have one minor concern. The subtitles from each section could be more informational. For example, some of the subtitles in the result section are exactly the same as the subtitles in the discussion sections with the same names such as "heading dates" . Please come up with some subtitles summarizing the take home messages or the most important conclusions for readers' convenience.

- *The subtitles are modified as suggested*
